# Including Distorted Specimens in Allometric Studies: Linear Mixed Models Account for Deformation

**DOI:** 10.1093/iob/obab017

**Published:** 2021-05-18

**Authors:** B M Wynd, J C Uyeda, S J Nesbitt

**Affiliations:** 1 Department of Geosciences, Virginia Tech, Blacksburg, VA 24061, USA; 2 Department of Biological Sciences, Virginia Tech, Blacksburg, VA 24061, USA

## Abstract

Allometry—patterns of relative change in body parts—is a staple for examining how clades exhibit scaling patterns representative of evolutionary constraint on phenotype, or quantifying patterns of ontogenetic growth within a species. Reconstructing allometries from ontogenetic series is one of the few methods available to reconstruct growth in fossil specimens. However, many fossil specimens are deformed (twisted, flattened, and displaced bones) during fossilization, changing their original morphology in unpredictable and sometimes undecipherable ways. To mitigate against post burial changes, paleontologists typically remove clearly distorted measurements from analyses. However, this can potentially remove evidence of individual variation and limits the number of samples amenable to study, which can negatively impact allometric reconstructions. Ordinary least squares (OLS) regression and major axis regression are common methods for estimating allometry, but they assume constant levels of residual variation across specimens, which is unlikely to be true when including both distorted and undistorted specimens. Alternatively, a generalized linear mixed model (GLMM) can attribute additional variation in a model (e.g., fixed or random effects). We performed a simulation study based on an empirical analysis of the extinct cynodont, *Exaeretodon argentinus*, to test the efficacy of a GLMM on allometric data. We found that GLMMs estimate the allometry using a full dataset better than simply using only non-distorted data. We apply our approach on two empirical datasets, cranial measurements of actual specimens of *E. argentinus* (*n* = 16) and femoral measurements of the dinosaur *Tawa hallae* (*n* = 26). Taken together, our study suggests that a GLMM is better able to reconstruct patterns of allometry over an OLS in datasets comprised of extinct forms and should be standard protocol for anyone using distorted specimens.

## Spanish

Alometria—el estudio de patrónes midiendo los cambios de proporciónes entre diferentes partes del cuerpo—es un método popularmente usado para estudiar como clados exhiben patrónes fenotípicos que representan restricciónes evolutivas, o para cuantificar patrónes de ontogenia entre una especie. Reconstruyendo alometrias para series ontogeneticas es uno de los pocos métodos disponibles para reconstruir el crecimiento de especies fósiles. Sin embargo, fósiles sufren de deformaciónes tafonomicas que alteran la morfología original y algunas veces en maneras no deseadas. Para mitigar estas alteraciones tafonomicas, paleontólogos excluyen mediciones alteradas de sus análisis. Desafortunadamente, esto limita el numero de muestras y potencialmente elimina evidencia de variación individual, impactando reconstrucciones alometricas. Mínimos Cuadrados Ordinarios (MCO) es un método frecuentemente usado para estimar alometria, pero asume niveles constantes de varición entre especímenes; esto es improbable cuando uno incluye especímenes deformados y especímenes indeformables. Alternativamente, Modelos Lineales Generalizados Mixtos (MLGM) pueden atribuir variciónes adicionales en un modelo. Nosotros corrimos simulaciones basadas en análisis empíricos del cinodonte extinto, *Exaeretodon argentinus*, para determinar la eficacia de MLGM con datos alometricos. Nosotros descubrimos que MLGM estima la alometria usando un conjunto de datos completos, en lugar de solo usar datos distorsionados. Aplicamos este método en dos conjuntos de datos empíricos: medidas craneales de especímenes de *E. argentinus* (*n* = 16) y medidas femorales del dinosaurio *Tawa hallae* (*n* = 26). Nuestros estudios indican que MLGM puede reconstruir mejor los patrónes de alometria sobre MCO con conjuntos de datos que incluyen especímenes extintos, y debería ser el protocolo estándar cuándo se usan especímenes que están deformados.

## Introduction

All living multicellular organisms grow and change through time, whether solely in absolute size or with changing proportions of individual features (Thompson 1917; Huxley 1932; Gould 1968, 1977; Gatsuk et al. 1980; Hochuli 2001; Rowe 2004; Tanner et al. 2010; Chagnon et al. 2013; Griffin et al. 2021). These patterns of growth can also influence evolutionary trajectories and allmetries among species, as they can reflect constraints on the variation available to evolutionary change (Gould 1966; Cheverud 1982; Alexander 1985; Klingenberg 1996a; Marroig and Cheverud 2005; Voje et al. 2014; Cardini 2019; Krone et al. 2019). Changes in the patterns of growth, or the study of allometry, has been explored extensively across the tree of Life (see Gould 1966), including extinct animals (e.g., Abdala and Giannini 2002; Kilbourne and Makovicky 2010; Jasinoski et al. 2015; Griffin and Nesbitt 2016a, 2016b; Krone et al. 2019). Allometric studies tend to focus on either identifying scaling patterns present in entire clades or reconstructing allometric patterns of a single population or species (Cheverud 1982; Pélabon et al. 2014; Klingenberg 2016).

Measuring allometric relationships is commonly used to characterize the likely evolutionary constraints of extant groups (Newell 1949; Marshall and Corruccini 1978; Radinsky 1984; Niklas 1994; Ungar 1998; Pyenson and Sponberg 2011; Boyer et al. 2013), as well as reconstructing patterns of growth in extinct species to better interpret their growth, taxonomy, and function (e.g., Abdala and Giannini 2000; Kilbourne and Makovicky 2010; Zhao et al. 2013; Foth et al. 2016; Chapelle et al. 2020). However, most fossils have been buried, undergoing post burial distortion which can cause bones to be compressed, twisted, and broken—which complicates their use in allometric studies (Behrensmeyer et al. 2000). Frequently, paleontologists are left with a mixture of well-preserved and distorted specimens.

Post-burial distortion is common, particularly in terrestrial fossils, where any deformation to the surrounding geologic environment has direct consequences on the fossil material. Post-burial deformation has often been grouped into one, or some combination, of crushing, shearing, breakage, displacement (via faulting), sub-burial abrasion, and erosion (e.g., Webster and Hughes 1999). Each of these modes of deformation may introduce variation into fossils through different means. Crushing, or compaction, may elongate gracile elements, while compacting and shortening more robust elements, or in the case of complexes of elements (e.g., a skull), crushing will often obscure surfaces from view entirely. Shearing will often introduce twisting, or sliding, of elements out of position, often represented as introducing a degree of asymmetry into bilaterally symmetrical elements (e.g., vertebrae or skulls). Displacement is often a consequence of compaction, wherein articulated elements are disarticulated and repositioned, but can also be caused via faulting, in which entire regions of a fossil will be shifted, and likely broken, to align with the degree of faulting in the surrounding rock unit. Finally, sub-burial abrasion and erosion produce similar results, with the destruction of fossil material, though erosion operates on a more extreme scale than sub-burial abrasion. Though, not an exhaustive list of taphonomic processes, each of these types of deformation introduces variation either in altering the shape, angle, or position of elements, or in removing information from a fossil that is otherwise necessary for analyses aimed at recovering biologic patterns.

For the cases in which the fossil material is left fully intact (e.g., shearing and light to moderate crushing), paleontologists have turned to retrodeformation analyses, in an attempt to return biological results from taphonomically altered materials. Largely utilized with geometric morphometrics (see Angielczyk and Sheets 2007; Schlager et al. 2018), these methods use either bilateral symmetry or morphology of a reference point (e.g., the orbit in Arbour and Currie 2012), to assign initial landmarks that are then retrodeformed to better reflect the original morphology in question and allow for comparison between specimens or species (Schlager et al. 2018). When complete skeletal models are necessary for analyses (e.g., finite element analysis), 3D processing software can be used to manipulate elements back into original biologic positions, given comparisons to exemplar specimens/modern correlates (Rayfield 2007; Arbour and Currie 2012; Cuff and Rayfield 2015; Lautenschlager 2016). These methods can be challenging to implement and may have biases (see Kammerer et al. 2020), but are extremely promising for the future of paleontological studies. However, such methods are not readily accessible and challenging to implement for the majority of current practitioners. In this contribution, we suggest that readily available statistical approaches are simple and straigthforward alternatives that can allow for usage of distorted data and can be widely-adopted to improve allometric coefficients from fossil data.

Measuring allometry will naturally depend on the definitions, assumptions, and mathematical models used (Alexander 1985). Herein, we focus on a regression-based approach that focus on the covariation among different traits (Huxley 1932; Jolicoeur 1963), as opposed to focusing on the covariation between size and shape (Mosimann 1970). The most often used methods to estimate the scaling of different features are the ordinary least squares (OLS) regression (hereafter called “linear regression”), and the reduced major axis analysis, which both aim to summarize linear relationships between variables (Gould 1966; Smith 2009). An alternative method is the multivariate allometry method (e.g., eigen analysis), which summarizes the greatest variation in variances and covariances between all variables simultaneously, using principal component analysis (Klingenberg 1996a; Klingenberg 2016). However, it is common practice to use models that assume the measurement data have identically distributed residual variation (i.e., the assumption of homoscedasticity). However, paleontological data often challenge researchers with specimens subject to additional sources of non-biologic variation (e.g., deformation), which violate this assumption by introducing an additional source of error for only a subset of observations. Consequently, while retrodeformation analyses is a promising solution (see above), the high demands of these approaches mean that more commonly researchers resort to removing perceived distorted measurements from analyses entirely, or including them despite the known uncertainty in their measurement (e.g., Abdala and Giannini 2000, 2002; Brown and Vavrek 2015). The former solution complicates the results of the analysis by removing potentially informative and hard to obtain data, while the latter adds variation unrepresentative of the sample as a whole. Given the often limited nature of fossil material, neither of these solutions are ideal. Ideally, our statistical tools should be able to incorporate additional sources of variation, so individual measurements and whole specimens do not have to be eliminated and maximal information can be extracted from our limited datasets.

In this article, we demonstrate the utility of a generalized linear mixed model (GLMM) in estimating allometry in fossil samples with clear distortion. We chose GLMMs because they follow the assumptions of linear models and can incorporate additional variation based on previously made observations as random effects (distortion) in the model (Bolker et al. 2009). We use measurements of fossilized specimens to simulate realistic datasets, adding additional variation in half the samples, and testing three different models: (1) linear regression of sample with no added variation, (2) linear regression of sample with added variation, and (3) GLMM of sample with added variation. We apply the approach on two fossil datasets, the crania of the Late Triassic cynodont, *Exaeretodon argentinus* (Cabrera 1943), and the femora of the Late Triassic dinosaur, *Tawa hallae* (Nesbitt et al. 2009), to demonstrate the effect of including both undistorted and distorted specimens analyzed with GLMMs.

## Materials and methods

### Simulation study

**Fig. 1:**
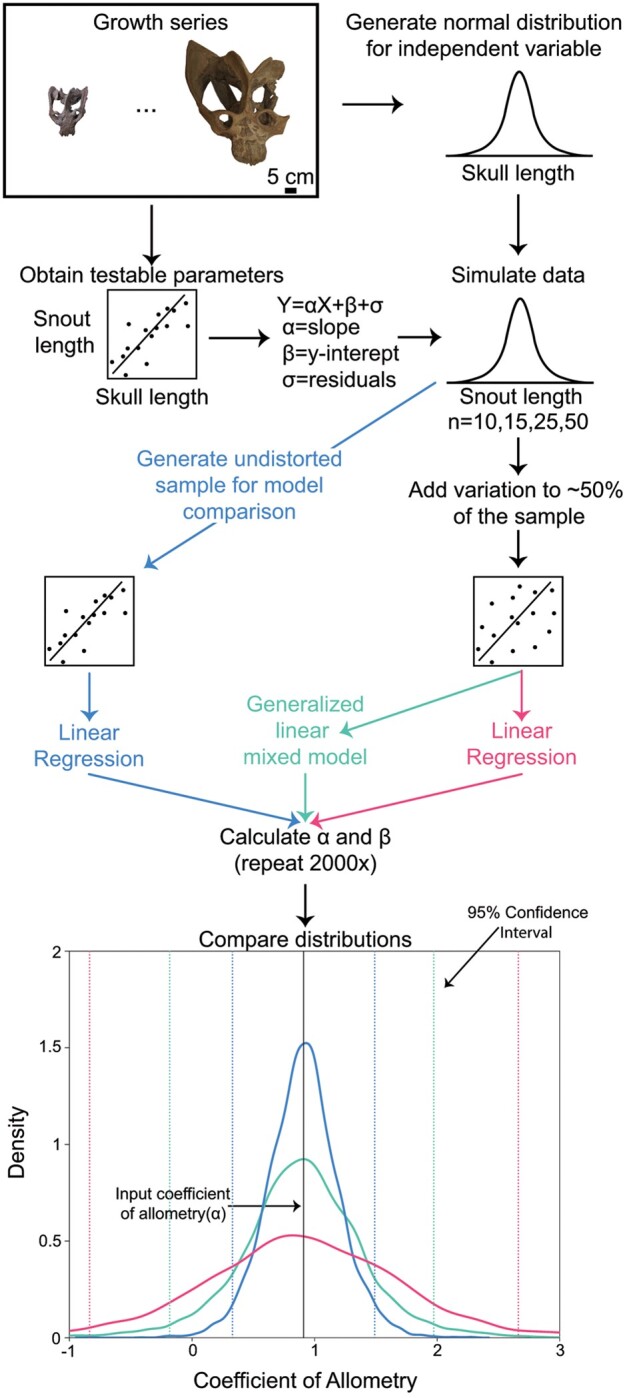
Flowchart depicting simulation methodology. Colors depicted here are reflected throughout the manuscript for the three different models. Distributions and scatterplots throughout the flowchart are not based on real data but visualization tools. The kernel density plot on the bottom is based on muzzle length in *E. argentinus*.

To assess the ability of GLMMs to recover allometric relationships, we built a simulation study based on cranial measurements from 16 specimens of the Late Triassic cynodont, *E. argentinus* (Fig. 1), 11 of which are accessioned at the Harvard Museum of Comparative Zoology (MCZ; Supplementary Table S1) and the rest are accessioned at the Colección de Paleontología de Vertebrados del Instituto Miguel Lillo (Supplementary Table S1). A linear regression model of skull length against snout length was performed on 15 of the 16 specimen sample (snout length is often robust and undistorted in *E. argentinus*, personal observation)—wherein eight measurements were scored as distorted *a priori*—using the lm command in the base R statistical environment (version 3.6, R Core Team 2018), to recover starting values of coefficient of allometry (= slope), *y*-intercept, and residual variation to be used in our simulation. We chose to use snout length for our empirical dataset because a GLMM of snout length returns no additional variation due to the random effect, fossilization (see below). As such, a regression of snout length reflects constant levels of variation across sample sizes (homoscedasticity) and is the most suitable measurement to assess how additional variation is modeled. The variance for the undistorted and distorted measurements of snout length is 0.021 and 0.019, respectively. Because the allometric equation is a power law equation, we log transform all measurements to fit them into a simple regression equation (Alexander 1985). We then built a normal distribution from the skull length measurements of our sample (logarithmic mean = 2.39, standard deviation = 0.14).

(1) Y= αX+ β+ ε.

We used the coefficient of allometry (*α* = 0.91), *y*-intercept (*β* = −0.37), and normally-distributed residual variation (ε, mean = 0.3, standard deviation = 0.1) from the regression analysis with the estimated skull lengths to generate four datasets consisting of 10, 15, 25, and 50 specimens. For each dataset, values of skull length (*X*) were sampled (*n* = 10, 15, 25, or 50) from our normal distribution and then were used in Equation (1) along with values for *y*-intercept and residual variation to generate estimated values for snout length (*Y*).

To include additional variation due to random effects (e.g., fossilization), we sampled a normal distribution (γ) with mean 0, and standard deviation 0.4 (on average, 1.5 times ε to simulate high variation due to fossilization). To ensure that the additional variation was not applied to all simulated measurements, we sampled a binomial distribution (*C*), with peaks at 0 (undistorted) and 1 (distorted), with a 50% sampling rate. Because mixed effects models require at least five to six blocks (i.e., individuals) per treatment (0 or 1; Bolker et al. 2009), the binomial distribution is unable to consistently produce sufficient analyzable samples at 10 specimens; to overcome this, we generated a vector with five observations of 0 and 1 each to ensure that the model will appropriately reconstruct allometry at sample sizes of 10 specimens. For the 15 specimen sample, we also generated a vector with five observations of 0 and 10 observations of 1, to test model efficacy with a largely distorted sample. We include this additional variation to Equation (1) to generate the equation

(2) Y=αX+β+ε+Cγ.

We implement this model in the R package *lme4* (Bates et al. 2007), such that *C* is coded as 0 or 1, and corresponds to distorted (1) or undistorted (0). This route allows us to sample a broad range of “distorted” specimens. When γ ∼ 0 and/or *C* = 0 (the specimen is undistorted), then the last term in Equation (2) adds no additional variation for that specimen.

To validate our estimated data, we ran a linear regression on the simulated data with no added variation, the results of which should closely match the input parameters. We then tested a simple linear regression model on the dataset that included the both original measurements and ∼50% that included additional variation (γ), the results of which should not closely match the input parameters. Finally, we tested a GLMM with one random effect on the dataset that includes additional variation. For each of the three models, we recovered coefficient of allometry (slope) and *y*-intercept values. We repeated simulations for each model 2000 times, and generated probability density curves for the estimated results to evaluate model performance. To assess the effect of sample size on model performance, we performed our simulation with four different sample sizes (10, 15, 25, and 50 specimens with ∼50% distorted specimens in each sample; Fig. 1). To evaluate differences between the coefficients of allometry returned for the linear regression on distorted and undistorted data versus the GLMM, we performed a paired *t*-test, which assesses overlap between two different distributions. To test the effects of the common practice of excluding distorted specimens relative to including them in our model, we compared how including all specimens (distorted and undistorted) in Equation (2) to only undistorted specimens in Equation (1). For each sample, any simulated measurements where *C* = 1, were not included and the remaining samples (*C* = 0) were tested using Equation (1). With this, we were able to compare the same data with and without the inclusion of additional variation due to fossilization, effectively testing the method that paleontologists have been using.

We only compared the GLMM with ordinary linear models, and did not compare with reduced major axis analysis or multivariate allometry (Klingenberg 1996a; Kilmer and Rodríguez 2017), which account for variation in both the *X* and *Y* parameters. More importantly, a reduced major axis analysis assumes that the *X* and *Y* parameters are independent of one another (symmetrical), such that the variation in *X* is not influenced by the variation in *Y* and vice versa (Smith 2009; Kilmer and Rodríguez 2017). Reduced major axis and linear regression are both useful methods, but they measure different things, and in regards to questions of allometry a linear regression is often more appropriate (see Hansen and Bartoszek 2012). Although incorporating our method in a multivariate allometry would be beneficial for studies including variance covariance matrices, it would require *a priori* estimations of the variation due to deformation and a novel method to incorporate that variation into the covariances, which is beyond the scope of this work.

### Empirical applications to *E. argentinus* and *T. hallae*

We tested two separate datasets, the crania of *E. argentinus* (*n* = 16, with 17 different cranial measurements), and the femora of *T. hallae* (*n* = 26, with five different femoral measurements). Specimens of *T. hallae* are accessioned at the Ruth Hall Museum of Paleontology (GR; Supplementary Table S1). For each measurement (e.g., snout length or femoral head width), we scored them as either distorted (*C* = 1) or undistorted (*C* = 0). We scored each measurement rather than individuals because the fossilization process does not uniformly distort specimens, and some regions of fossils can retain more morphological integrity than others. For example, the snout region of *E. argentinus* is a complex of thick, tightly sutured bones forming a strong, dense, integrated structure, whereas the braincase is made up of thin bony walls and few supporting structures, resulting in many skulls with intact snouts but distorted braincases. No measurements were taken for features that were broken or those where the features were not visible; for example, many skulls of *E. argentinus* are still encased in plaster, such that the dorsal surface of the skulls are not presently available. Importantly, total length is often not greatly distorted in either *E. argentinus* or *T. hallae*, such that we expect low additional variation due to fossilization in estimations of *X* (see Table 1).

**Table 1:** Sample size and variance for fossil datasets

Taxon	Feature	Number of distorted	Variance distorted	Number of undistorted	Variance undistorted	Number of total
*Exaeretodon*	Skull length	0	0	16	0.019	16
*Exaeretodon*	Snout length	8	0.019	7	0.021	15
*Tawa*	Femur length	0	0	26	0.009	26
*Tawa*	Femoral head length	10	0.016	16	0.01	26

We performed a Shapiro–Wilk test on each measurement for each taxon to evaluate whether or not the input data were normally distributed. For *E. argentinus*, the palate length and upper post-canine length were marginally significant (*P* < 0.1), whereas orbit length and diastema length were significant (*P* < 0.05), suggesting that these data are non-normally distributed. For *T. hallae* only the minimum midshaft diameter was significant (*P* = 0.047) indicating a non-normal distribution for these data. We performed a Shapiro–Wilk test on all of the distorted-only measurements and found that all follow normal distributions, with the exception of skull width and transverse process width in *E. argentinus* which were marginally significant (*P* < 0.1), and basicranial length and diastema length in *E. argentinus* which were significantly different from normality (*P* < 0.05). The distorted measurements for upper post-canine length and zygoma height in *E. argentinus* lacked the necessary sample size to perform a Shapiro–Wilk test. We focus herein on snout length in *E. argentinus* and femoral head length in *T. hallae* (see supplement for analyses of additional measurements; Supplementary Table S2), which have 15 specimens with 8 distorted measurements and 26 specimens and 10 distorted measurements, respectively.

**Fig. 2:**
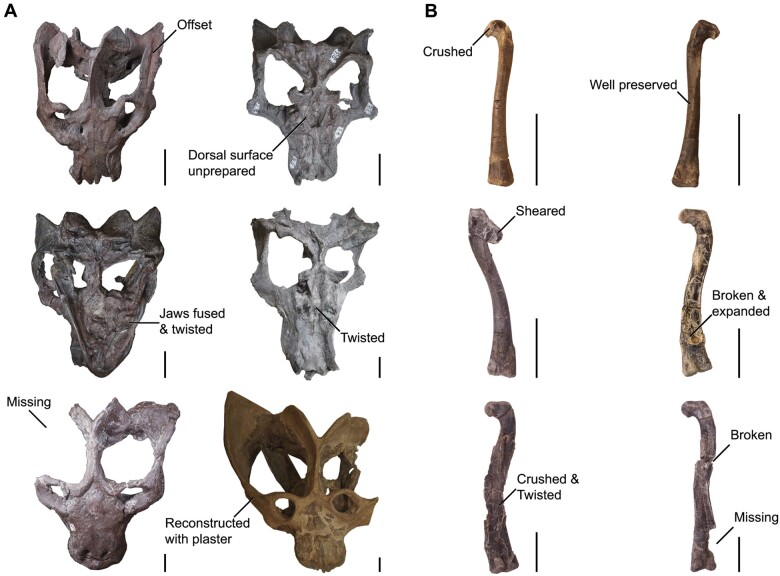
Varying degrees of deformation in samples of (**A**) *E. argentinus* (top row MCZ VPRA-4470 and MCZ VPRA-4472; middle row MCZ VPRA-4493 and MCZ VPRA-4468; bottom row MCZ VPRA-4505 and MCZ VPRA-4486) and (**B**) *T. hallae* (top row GR 244; middle row GR 578 and GR 226; bottom row GR 1050 and GR 1043). Specimen numbers follow left to right. Scale bars equal 5 cm.

To further assess model performance on specimen data given possible non-normality, we performed a non-parametric bootstrap analysis using two fossil datasets (Fig. 2). A non-parametric bootstrap resamples the data with replacement to assess model error, effectively testing how the model would respond if some of the data were left out. We chose a non-parametric bootstrap to assess model error and the effect of sample size on results. We ran the non-parametric bootstrap analyses on the two datasets using three different models: linear regression on only undistorted specimens (*C* = 0), linear regression on the full sample (distorted and undistorted specimens), and GLMM on the full sample (distorted and undistorted specimens); just as in the simulation study, to compare the effects of model and data on estimation of model parameters. For 5000 bootstrap iterations, we examined density distributions of coefficient of allometry and compare the 95% confidence intervals across models. We also compared intercept, but primarily focus on the coefficient of allometry as it is the most meaningful parameter for most allometric studies. Following the bootstrap analysis, we performed a likelihood ratio test with a chi-squared test (Stram and Lee 1994), to compare Equation (2) to a GLMM where variation is allowed in both *X* and *Y*, testing whether or not variation in slope has significant effects on reconstructed patterns.

For the GLMM, we follow Equation (2), where *ε* is random variation that is interpreted here as being produced by the fossilization process. We estimate fossilization as a random effect, because across all of our samples, we do not expect fossilization to consistently produce the same amount of variation across samples. However, if deformation is expected to have a consistent effect, it could be investigated as a fixed effect or be fit in a multiple linear regression. With this, *C*γ represents random error that only applies to distorted specimens, where *C* = 1. An important caveat to this model is that it requires at least five individual blocks (specimens) in each of the two categories to reliably reconstruct the random effects (Bolker et al. 2009). Because of this, the GLMM is not suitable for small datasets (*n* < 10). However, the model is conservative, and when the model finds no random variation, even with regions coded as distorted, it returns a linear model with ∼0 variance allocated to the additional parameter *C*γ (see Supplementary Table S3), indicating that the model will not underestimate the residuals when distorted measurements are included as random effects. This allows researchers to be cautious in scoring their specimens and still recover results representative of the biology.

## Results

### Simulation study

**Fig. 3:**
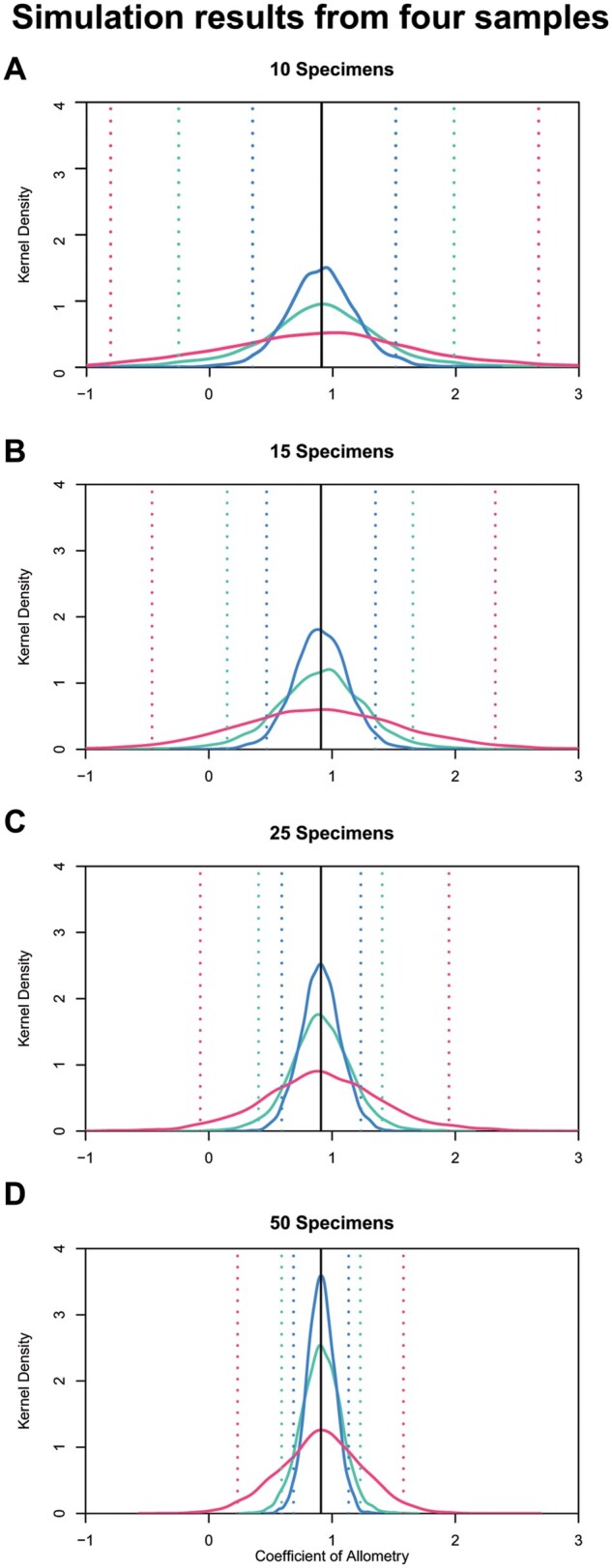
Simulation results of four different sample sizes where the linear regression on undistorted data consistently performs best, followed by the GLMM. GLMM is tested on a dataset with ∼50% of each dataset having additional variation. Dotted lines represent the upper and lower 95% confidence intervals for each model. (**A**) 10 specimens, (**B**) 15 specimens, (**C**) 25 specimens, and (**D**) 50 specimens.

**Fig. 4:**
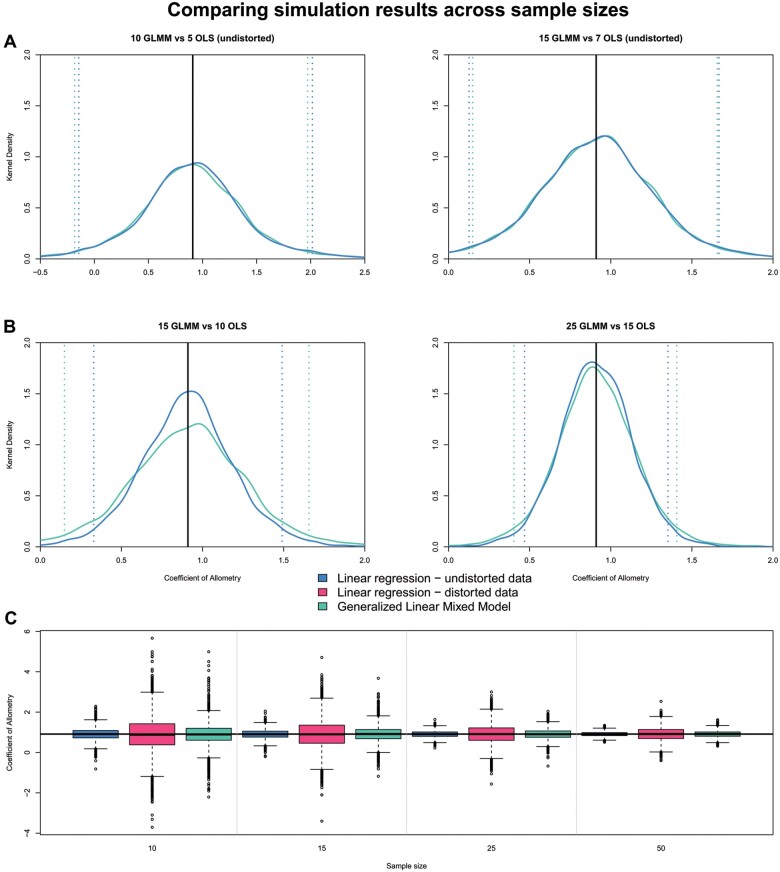
Simulation results comparing across different sample sizes, showing close similarity between GLMM and linear regression. GLMM is tested on a dataset with ∼50% of each dataset having additional variation. Dotted lines represent the upper and lower 95% confidence intervals for each model. (**A**) Fifteen specimens under a GLMM compared to 10 specimens from a linear regression. (**B**) Twenty-five specimens under a GLMM compared to 15 specimens from a linear regression. (**C**) Box plot representing all of the returned distributions for the three different models.

For each of the sample sizes, the linear regression on the undistorted sample (with no added variation) consistently performed best, based on the most frequent outcome at a mean coefficient of allometry of 0.91 (= true mean for simulation) and the lowest 95% confidence intervals (Fig. 3). This is to be expected, as the linear regression on the undistorted set acts as our benchmark for parameter estimation under an idealized dataset. For each sample size, the mixed effects model consistently estimated the slope closer to the expected mean (=0.91), than a linear regression that included distorted specimens. To test if the results were biased, we performed a paired *t*-test between the GLMM and the linear regression on the undistorted dataset across all simulated sample sizes, and found no significant difference (*n* = 10, *t* = 0.92, *P* = 0.36; *n* = 15, *t* = −0.96, *P* = 0.33; *n* = 25, *t* = 0.17, *P* = 0.86; *n* = 50, *t* = −0.02, *P* = 0.99). To assess how well the GLMM model compared with a control, we compared the distorted GLMM with the linear regression of only undistorted data at differing sample sizes. We found that a sample of 25 specimens—∼50% of which are scored as distorted—in the GLMM model closely overlapped with the kernel density plot of a linear regression of 15 undistorted specimens (Fig. 4A). The overlap was less profound in comparing kernel density plots of 15 distorted specimens (GLMM) to 10 undistorted specimens (linear regression), but they still share a clear peak at the expected mean value (Fig. 4B). Looking at the entire distribution of data (Fig. 4C), the distorted datasets always perform worse than the undistorted data, as expected. However, the GLMM consistently outperforms the linear regression on the same full dataset (both distorted and undistorted measurements).

### Non-parametric bootstrap

**Fig. 5:**
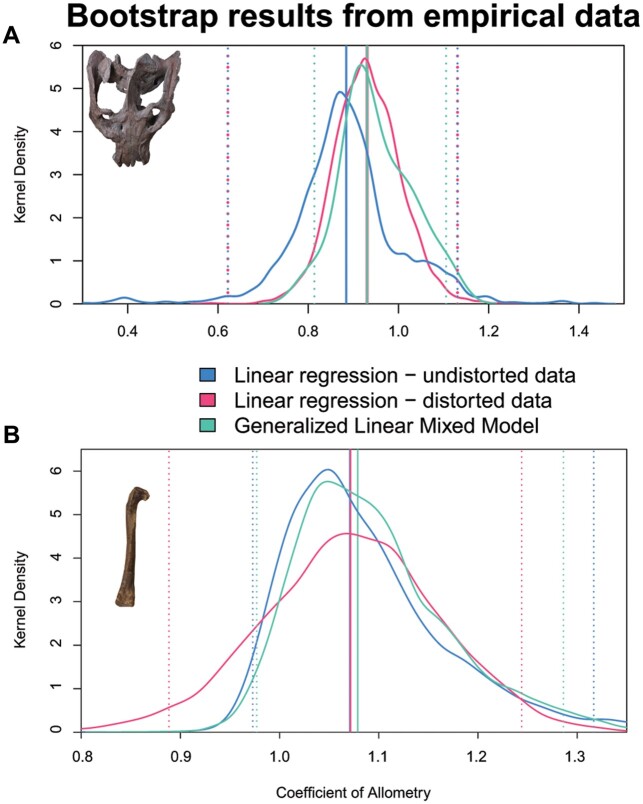
Bootstrap results for samples from (**A**) *E. argentinus* muzzle versus skull length and (**B**) *T. hallae* femoral head length versus femoral length. Each plot shows strong overlap between the returned distributions suggesting overall similarity in error. Dotted lines represent the upper and lower 95% confidence intervals for each model. Coefficient of allometry is more similar in the *T. hallae* sample than the *E. argentinus* sample, which is to be expected given the difference in sample size (*E. argentinus n* = 15; *T. hallae n *= 26). Skull is specimen MCZ VPRA-4470 and femur is specimen GR 244.

**Fig. 6:**
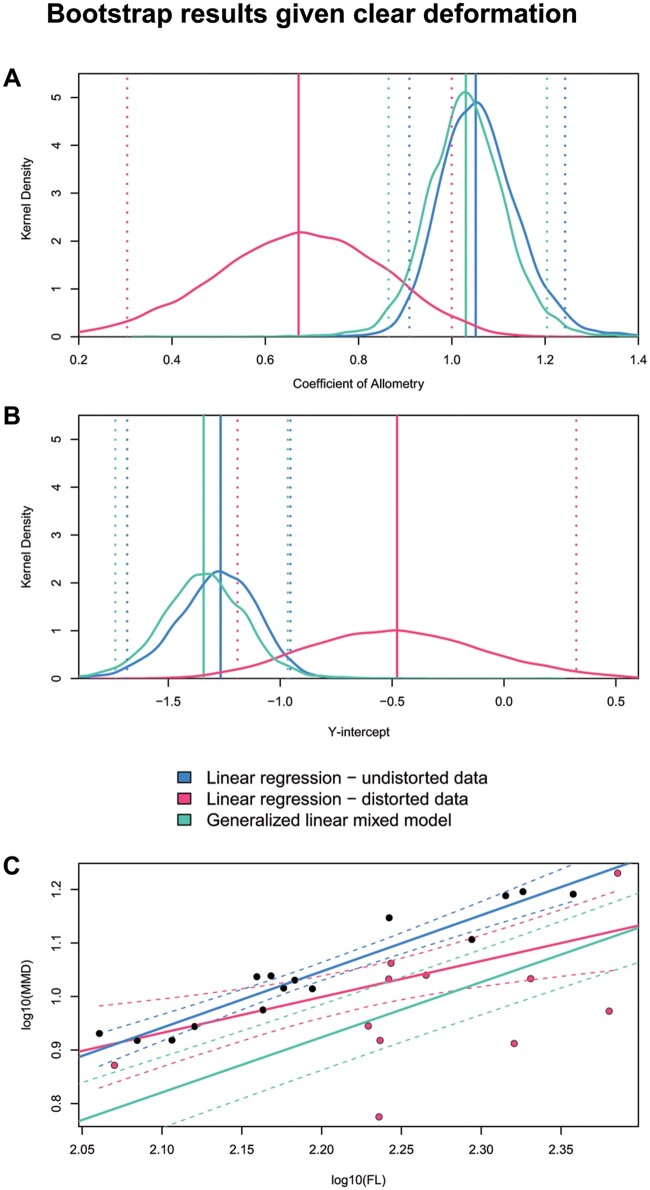
GLMM closely approximates the truth when significant deformation is introduced into the model for a sample of *T. hallae*. (**A**) Slope and (**B**) *y*-intercept suggest high similarity between the results of the linear regression on undistorted data and the GLMM. However, panel (**C**) shows that the inclusion of undistorted specimens (black dots) with a linear regression on only these undistorted specimens (blue line), distinctly crushed specimens (red circles) in a linear regression (red line), and the GLMM has a significant effect on recovering the intercept. Dotted lines represent the upper and lower 95% confidence intervals for each model. The *y*-intercepts betweenthe linear regression on undistorted data and the GLMM suggest that one should not heavily weigh interpretations based on *y*-intercept returned from a GLMM. Femur is specimen GR 244.

For both the *E. argentinus* and *T. hallae* datasets, across all features, the linear regression on the undistorted data produced density peaks with the narrowest 95% confidence intervals (Fig. 5). For the majority of *T. hallae* and *E. argentinus* measurements, the density distribution for coefficient of allometry closely followed the patterns of the linear regression on the undistorted sample (see Supplementary Figs. S1–S6). The bootstrap analysis using the GLMM on both undistorted and distorted specimens frequently recovered coefficients of allometry that were distinct from the other two models. Our chi-squared test between GLMMs both with and without variation in *Y* found no significant difference (chi-squared = 0.86, *P* > 0.95), which indicates that accounting for variation in *Y* is not necessary for these studies. A bootstrap of femoral midshaft diameter against total length in *T. hallae* shows that the regression of undistorted specimens and the GLMM closely mirror one another around isometry to slightly positive allometry, whereas the linear model on the full dataset recovers a pattern of distinctly negative allometry (Fig. 6A). Similar results are reported for the recovered *y*-intercept values (Fig. 6B); however, the GLMM and regression on undistorted data do not converge on the median intercept (regression = −1.26; GLMM = −1.34) which can have downstream consequences for extrapolating data from these models (log 10 difference = 0.072; retrotransformed differenced = 0.0082 mm; see Fig. 6C). These reconstructed *y*-intercepts produce minor differences in estimating feature sizes (with slope constant) when the independent variable (femur length here), is relatively small; for example, estimating midshaft diameter on a 100 mm femur produces a minimum midshaft diameter of 5.82 and 6.89 mm for the linear regression and GLMM *y*-intercepts, respectively. In this example, the *y*-intercept from the linear regression reconstructs femur length at 84.8% the size of the *y*-intercept from the GLMM, which can lead to distinct estimations of feature size when femur length is large (e.g., >200 mm). A logarithmic plot of femur length against midshaft diameter reveals a cluster of six specimens that are distinctly offset from the regression line and were coded as distorted *a priori* (Fig. 6C), indicating strong deformation that is accounted for in the GLMM. We sample basicranial length and palate length in *E. argentinus* (see Supplementary Figs. S2 and S4), neither of which have a distorted sample that meets minimum block size (*n* ≥ 5). Together, they illustrate that the GLMM will default to an OLS regression when confronted with small datasets (Supplementary Fig. S2), or, if there is considerable variance in the undistorted sample, both the GLMM and OLS regression will struggle to optimize a single peak for either coefficient of allometry or *y*-intercept (Supplementary Fig. S4). Although the GLMM always has slightly greater model error than the linear regression on undistorted data, we find support for its efficacy in reconstructing allometry in specimens that exhibit deformation.

## Discussion

### Simulations appropriately incorporate additional variation

Our simulations of datasets with distorted fossils suggest that a GLMM is able to account for additional sources of variation when reconstructing allometric relationships. As expected, a sample of only undistorted data (i.e., no additional variation) consistently produced density distributions closest to the true value and acted as a benchmark for each of our selected sample sizes. The GLMM consistently produced density distributions that more closely followed the sample of only undistorted data than the linear regression on the dataset with undistorted and distorted measurements (i.e., with additional variation), such that trials returned lower overall variance in the allometric parameters (slope, intercept, and residuals) and higher density around the input parameters. These results suggest that a GLMM can account for unknown sources of variation in a sample, making such a test amenable to fossil samples that include considerable variation in preservation state (Abdala and Giannini 2000; Kammerer et al. 2012; Wang et al. 2017). However, a GLMM should not be used with small sample sizes (*n* < 10) or those that have few distorted or undistorted measurements, as GLMM’s fail to infer random effects where there are fewer than five to six specimens for the random effects to understand the distinction between distorted and undistorted in the sample (Bolker et al. 2009). Though this is a challenge for many datasets (particularly paleontological datasets), the *lme4* package is forgiving to the cautious observer, such that it will not attribute variation to external samples when there is none (see Supplementary Table S3). If the GLMM finds that all of the datapoints lie within the residuals of the regression, then it will assign no additional variation to the random effect and will simply return the results of a linear regression. Our simulation results indicate that a GLMM is a viable replacement for a linear regression model when there are sources of unknown variation throughout a sample that would otherwise preclude those specimens from the study. Furthermore, including the full dataset can give better approximations of the parameters of interest, and the individual variation that is representative of the full sample (see Brown and Vavrek 2015).

### Linear mixed effects models can estimate allometric parameters

We tested the GLMM with allometric data to estimate its efficacy as a replacement for OLS regression given fossil datasets. The results of our simulation suggest that the GLMM closely approximates the input parameters, and has peak densities lower than, but close to our control of a linear regression on undistorted data (Figs. 3 and 4). This suggests that while not as consistent as the undistorted dataset, the GLMM has an overall variance and precision similar to the linear regression on undistorted data, and has considerably lower variance in returned parameter estimates than the linear regression on the distorted dataset, indicating that it can accurately reconstruct allometric parameters. For any studies that would include marginally distorted measurements or estimations of measurements, an OLS approach with few specimens could greatly affect any returned parameters. Therefore, it is important to use a GLMM on samples that would have otherwise been forced into an OLS model and treated as if they were undistorted. Furthermore, the deviation from the input parameters is relatively small in observing allometric patterns, such that one would not expect to see a negative allometric signal with the linear regression versus a positive allometric signal with the GLMM. One may see deviations from isometry based on model choice; however, this would likely occur when sample sizes are low (≤5) for undistorted specimens, and the residual variation would indicate that neither model is significantly different from isometry (see Supplementary Figs. S1–S7). Although this simulation suggests that GLMMs can be used in estimating allometric and isometric patterns given additional sources of variation, it does not address model efficacy when confronted with actual data.

Our bootstrap analysis tests efficacy between distinct models by estimating the error present in the data, which is largely reflective of sample size, and comparing the overlap in the distributions of returned traits (Hinkley 1988), such as coefficient of allometry or *y*-intercept. The bootstrap analyses on the *T. hallae* samples (see Fig. 5) suggest that the GLMM results have clear overlap with the results from the linear regression on only undistorted measurements (i.e., control), but their peaks do not directly overlap one another. This is to be expected, as many of the distorted specimens in the *T. hallae* sample are clustered near our smallest and largest samples, and thus including these specimens will influence the slope and as a result, the intercept value for these parameters. Furthermore, the GLMM consistently produces results with narrower 95% confidence intervals than the linear regression on the complete dataset, suggesting that it is appropriately accounting for additional sources of variation, given real data. The results of the *E. argentinus* sample share consistencies with the *T. hallae* dataset, but also show greater variance in how much overlap exists between the GLMM and the linear regression on undistorted measurements. This is due to sample size and preservation in *E. argentinus*, where we only have access to measurements from 16 specimens, and many of them show clear deformation or breakage in and around the braincase. Because of this, bootstrap replicates have high variance in regression parameters. However, even with this, multiple features (see Supplementary Figs. S2, S5, and S6) with closely overlapping parameter distributions between the GLMM and the linear regression on undistorted datasets, suggesting that given enough samples (10–15 specimens), the GLMM is a suitable substitution for a linear regression. Taken together, the results of our analyses suggest that a GLMM can reconstruct allometric relationships in cases where distorted specimens result in heteroscedasticity. Given any deformation in a sample, the GLMM is an appropriate model to estimate additional variance without removing specimens or introducing individual biases (Bolker et al. 2009).

These analyses carry a simple assumption, that variation during fossilization is non-directional, with no discernable pattern between specimens. This suggests, variation should not consistently bias a sample toward one end of the distribution. This assumption is clearly violated in the case for minimum midshaft diameter in *T. hallae* (see Fig. 6B), where variation consistently results in underestimating the midshaft diameter of the femur. Importantly, this can be modeled into a GLMM as a fixed effect (Bolker et al. 2009), indicating that these specimens should produce a consistent directional effect on parameter estimations. The degree of distortion, and consistency in the types of distortion, must be addressed *a priori* to assess whether the patterns appear to be directional or random. GLMMs are versatile models that can be tailored to the data, using prior information, to most appropriately estimate allometric patterns given differing forms of distortion (see Bolker et al. 2009), and thus should be further explored by the paleontological community.

### Individual variation, post-burial distortion, growth trends, and their importance

Allometric relationships have been used extensively to study the how patterns of divergence in trait-scaling relationships (i.e., the evolutionary allometry) can be related to variation at the individual (ontogenetic) or population level (Cock 1966; Lande 1979; Klingenberg 1996b; Klingenberg et al. 2001; Marroig and Cheverud 2005; Griffin and Nesbitt 2016b; Cardini 2019). These distributions can only be understood when sampling a wide breadth of individuals, where each individual defines a distinct point on any plot (Brown and Vavrek 2015), which is often a challenge for paleontological studies. For studies of allometry or any linear relationship within a species or population, every specimen for an analysis is informative and useful, and while not a major issue for many easily-obtained extant species, this is often insurmountable for those working with fossil specimens. Not only are the number of individuals limited, but they are often incomplete, broken, or misshapen, making any measurements non-reflective of the morphology of the organism during life. The remedies to this have been to estimate the distorted measurements or to simply leave them out of the analysis, sacrificing individual variation in the process. We show here that no specimens need to be removed in these analyses, and furthermore, our simulations reveal that a mix of 15 distorted and undistorted specimens is often a stronger sample than 10 undistorted specimens, when the right model is employed. We find that a GLMM is able to estimate additional variation, reconstruct allometric relationships, and retain the critical individual variation in studies of allometry in specimens showing any degree of distortion. We recommend that either retrodeformation or statistical techniques that account for distortion should be used, rather than sacrificing the precious data that we have.

## Author’s contributions

B.M.W., J.C.U., and S.J.N. conceived and directed the study and edited the manuscript. B.M.W. collected the data, performed the analyses, and wrote the first draft of the manuscript.

## Supplementary Material

obab017_Supplementary_Data
