# Supplementary material for: Including Distorted Specimens in Allometric Studies: Linear Mixed Models Account for Deformation
Source: Integr Org Biol. 2021 May 18;3(1):obab017. doi: 10.1093/iob/obab017 (PMC8341891; doi:10.1093/iob/obab017)
Supplement: obab017_Supplementary_Data [file obab017_supplementary_data.zip › obab017_Supplementary_Data/Wynd et al supplement_R1.docx]

SUPPLEMENT TO:
INCLUDING DISTORTED SPECIMENS IN ALLOMETRY: USING LINEAR MIXED MODELS TO ACCOUNT FOR DEFORMATION

BRENEN M. WYND^*,1^, JOSEF C. UYEDA^2^, and STERLING J. NESBITT^1^

^1^Department of Geosciences, Virginia Tech, Blacksburg, VA 24061, U. S. A., [bmwynd@vt.edu](mailto:bmwynd@vt.edu); [sjn2104@vt.edu](mailto:sjn2104@vt.edu)

^2^Department of Biological Sciences, Virginia Tech, Blacksburg, VA 24061, U. S. A., [juyeda@vt.edu](mailto:juyeda@vt.edu)

**SPECIMENS**

Specimen measurements for all *Tawa* and MCZ specimens were taken by BMW using a pair of digital calipers accurate to 0.01 mm (see Table S1). Measurements for all PVL specimens were taken by Fernando Abdala and shared with BMW for a separate project (see Table S1).

**Institution abbreviations**

**GR**, Ruth Hall Museum of Paleontology at Ghost Ranch, New Mexico, USA; **MCZ VPRA**, The Louis Agassiz Museum of Comparative Zoology, Cambridge, Massachusetts, USA; **PVL**, Colección de Paleontología de Vertebrados del Instituto Miguel Lillo, San Miguel de Tucumán, Argentina.

TABLE S1: Specimens and elements used in bootstrapping analysis.

| **Specimen number** | **Taxon name** | **Element** |
| --- | --- | --- |
| GR 226 | *Tawa hallae* | Right femur |
| GR 235 | *Tawa hallae* | Right femur |
| GR 244 | *Tawa hallae* | Left femur |
| GR 355 | *Tawa hallae* | Right femur |
| GR 405 | *Tawa hallae* | Left femur |
| GR 455 | *Tawa hallae* | Left femur |
| GR 463 | *Tawa hallae* | Right femur |
| GR 480 | *Tawa hallae* | Right femur |
| GR 502 | *Tawa hallae* | Left femur |
| GR 504 | *Tawa hallae* | Right femur |
| GR 578 | *Tawa hallae* | Left femur |
| GR 1040 | *Tawa hallae* | Right femur |
| GR 1042 | *Tawa hallae* | Left femur |
| GR 1043 | *Tawa hallae* | Left femur |
| GR 1044 | *Tawa hallae* | Right femur |
| GR 1045 | *Tawa hallae* | Left femur |
| GR 1046 | *Tawa hallae* | Left and right femora |
| GR 1047 | *Tawa hallae* | Left femur |
| GR 1048 | *Tawa hallae* | Left femur |
| GR 1049 | *Tawa hallae* | Right femur |
| GR 1050 | *Tawa hallae* | Right femur |
| GR 1051 | *Chindesaurus* sp. | Left femur |
| GR 1054 | *Tawa hallae* | Left femur |
| GR 1058 | *Chindesaurus* sp. | Right femur |
| GR 1060 | *Chindesaurus* sp. | Left femur |
| GR 1061 | *Tawa hallae* | Left femur |
| GR 1062 | *Chindesaurus* sp. | Right femur |
| MCZ VPRA-338-58M | *Exaeretodon argentinus* | Skull |
| MCZ VPRA-4468 | *Exaeretodon argentinus* | Skull |
| MCZ VPRA-4470 | *Exaeretodon argentinus* | Skull |
| MCZ VPRA-4472 | *Exaeretodon argentinus* | Skull |
| MCZ VPRA-4478 | *Exaeretodon argentinus* | Skull |
| MCZ VPRA-4483 | *Exaeretodon argentinus* | Skull |
| MCZ VPRA-4486 | *Exaeretodon argentinus* | Skull |
| MCZ VPRA-4493 | *Exaeretodon argentinus* | Skull |
| MCZ VPRA-4494 | *Exaeretodon argentinus* | Skull |
| MCZ VPRA-4505 | *Exaeretodon argentinus* | Skull |
| MCZ VPRA-4781 | *Exaeretodon argentinus* | Skull |
| PVL 2056 | *Exaeretodon argentinus* | Skull |
| PVL 2082 | *Exaeretodon argentinus* | Skull |
| PVL 2094 | *Exaeretodon argentinus* | Skull |
| PVL 2473 | *Exaeretodon argentinus* | Skull |
| PVL 2565 | *Exaeretodon argentinus* | Skull |

**ADDITIONAL BOOTSTRAP RESULTS**

We include the density distributions for all of the other measurements that were amenable to bootstrap analysis. We do not include plots that were unable to reconstruct density distributions for all three of our trials (ordinary least squares regression on undistorted data, ordinary least squares regression on distorted data, generalized linear mixed model on distorted data), as for many of our lower sample sizes (n < 13) we were unable to recover density distributions for coefficient of allometry (slope) under the generalized linear mixed model. For each of our following plots the generalized linear mixed model is shown in gold, the ordinary least squares regression on distorted data is shown in red, and the ordinary least squares regression is shown in blue, as it is in the main paper. We also include code that outlines our simulation study, and our bootstrap analysis (Supplementary data 1). We include raw measurements (not log transformed) for *Tawa hallae*, but not for *Exaeretodon argentinus* (Supplementary data 2). We do not include the *E. argentinus* raw data because these data are being used in a currently unpublished separate project that is reconstructing patterns of ontogenetic allometry in *E. argentinus*. For the majority of the *E argentinus* data we recover wide error distributions from bootstrap analyses where the generalized linear mixed model frequently does not coincide with the density distributions for the ordinary least squares regression on undistorted data. This is largely because of the low sample sizes in *E. argentinus* and the roughly equal number of distorted and undistorted specimens for each individual measurement. When the model lacks an appropriate number of individuals in each of the two groups (~5), it returns results for an ordinary least squares regression. Ultimately, we find that the generalized linear mixed model is able to approximate allometric results given adequate sample sizes of both distorted and undistorted measurements. We include palate and basicranial length to illustrate the effects of small sample size (distorted sample < 5 specimens). Palate length shows multiple peaks, reflecting the wide distribution of input data and the poor convergence on a linear pattern that is representative of the group. Whereas basicranial length shows a pattern that is roughly equivalent to the linear regression of undistorted data, reflecting the fact that the GLMM returns an ordinary least squares regression when sample sizes are not adequate. Additionally, we show the model output for the generalized linear mixed model on muzzle length in *E. argentinus*, where we find extremely low variance in the random effect of distortion, essentially indicating that the muzzle does not reflect an appreciable amount of distortion (Table S3).

Table S2: Sample size and variance for reported measurements

| Taxon | Feature | # distorted | Variance distorted | # undistorted | Variance undistorted | # total |
| --- | --- | --- | --- | --- | --- | --- |
| *Exaeretodon* | Basicranial length | 3 | 0.005 | 8 | 0.014 | 11 |
| *Exaeretodon* | Orbit length | 6 | 0.025 | 7 | 0.004 | 13 |
| *Exaeretodon* | Palate length | 7 | 0.019 | 3 | 0.028 | 10 |
| *Exaeretodon* | Skull width | 8 | 0.024 | 6 | 0.018 | 14 |
| *Exaeretodon* | Temporal length | 5 | 0.023 | 8 | 0.026 | 13 |
| *Tawa* | Femoral head width | 7 | 0.033 | 19 | 0.007 | 26 |


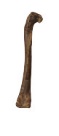

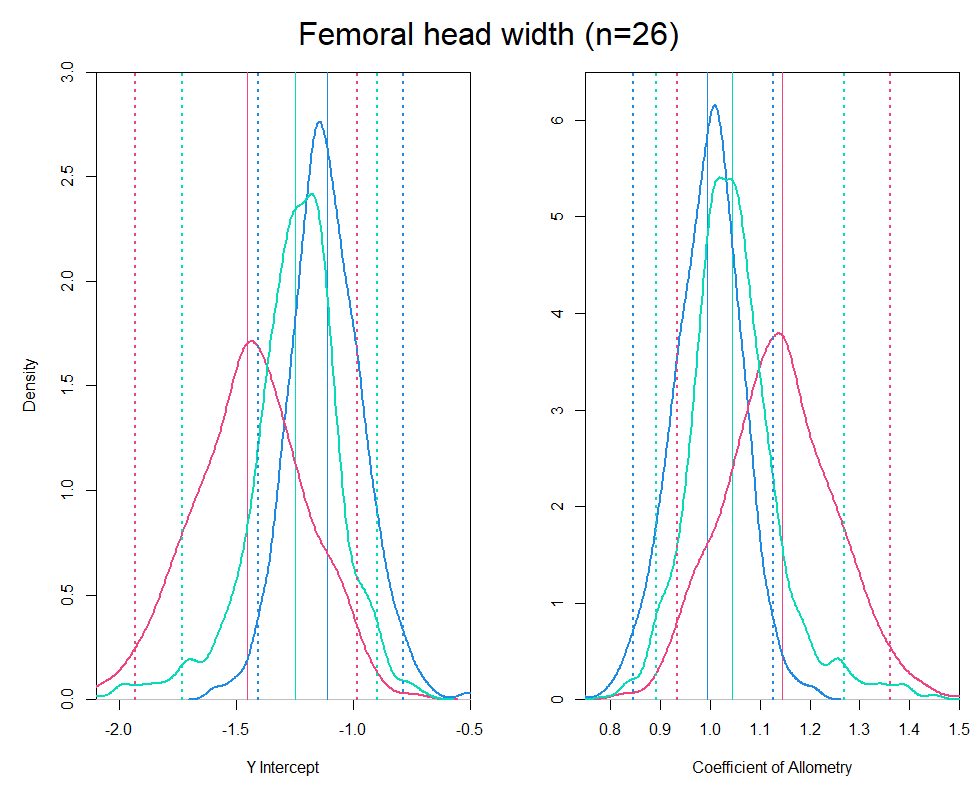
**Fig S1**: Bootstrapping femoral head width versus total femur length in *Tawa hallae*. Cyan=generalized linear mixed model; Blue=ordinary least squares regression on undistorted data; Red=ordinary least squares regression on distorted data; and Dashed lines=95% confidence intervals; solid lines=recovered y-intercept and coefficient of allometry from analyses on the full dataset. Femur is specimen GR 244.


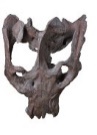

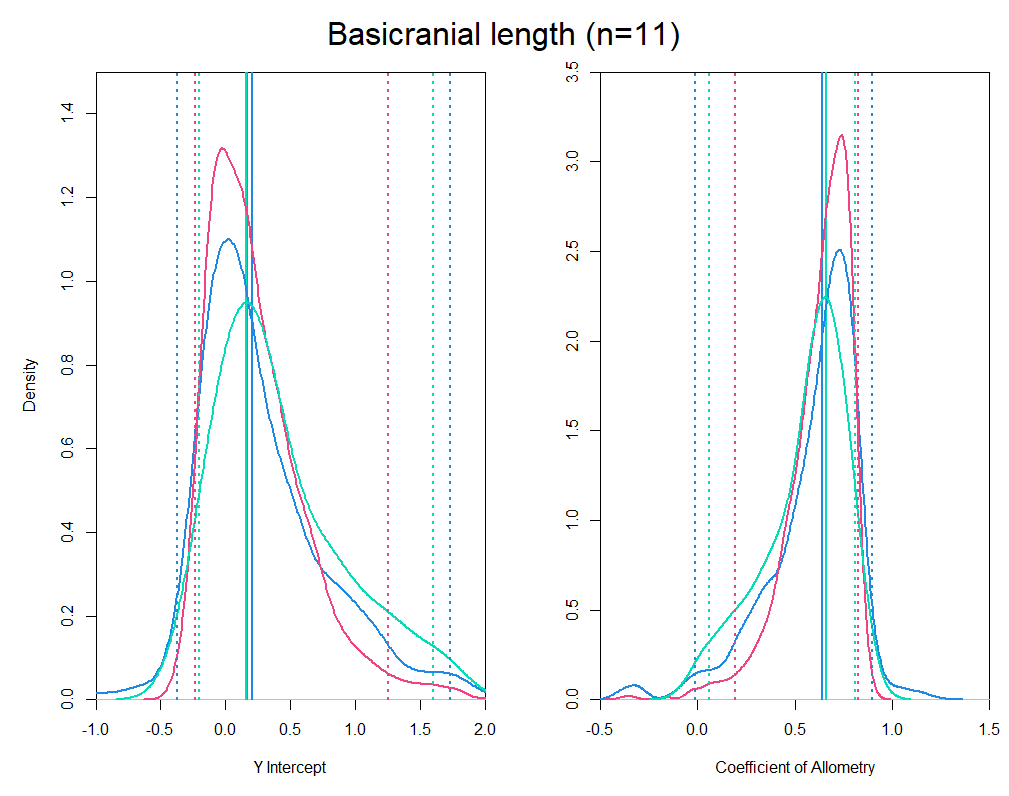


**Fig S2:** Bootstrapping basicranial length against total skull length in *Exaeretodon argentinus*. Cyan=generalized linear mixed model; Blue=ordinary least squares regression on undistorted data; Red=ordinary least squares regression on distorted data; and Dashed lines=95% confidence intervals; solid lines=recovered y-intercept and coefficient of allometry from analyses on the full dataset. Skull is specimen MCZ VPRA-4470.


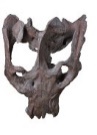

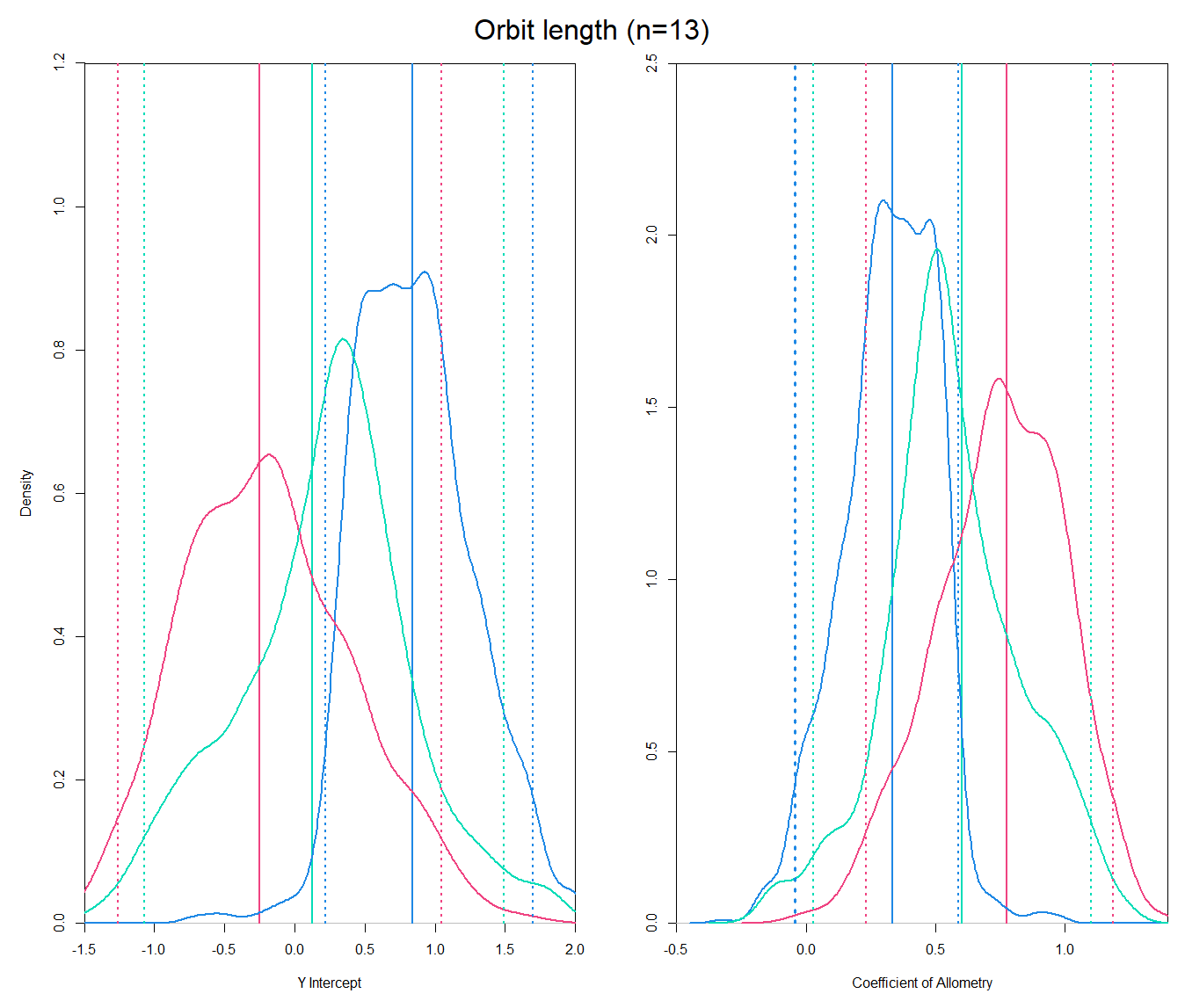


**Fig S3:** Bootstrapping orbit length against total skull length in *Exaeretodon argentinus*. Cyan=generalized linear mixed model; Blue=ordinary least squares regression on undistorted data; Red=ordinary least squares regression on distorted data; and Dashed lines=95% confidence intervals; solid lines=recovered y-intercept and coefficient of allometry from analyses on the full dataset. Skull is specimen MCZ VPRA-4470.


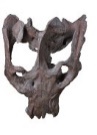

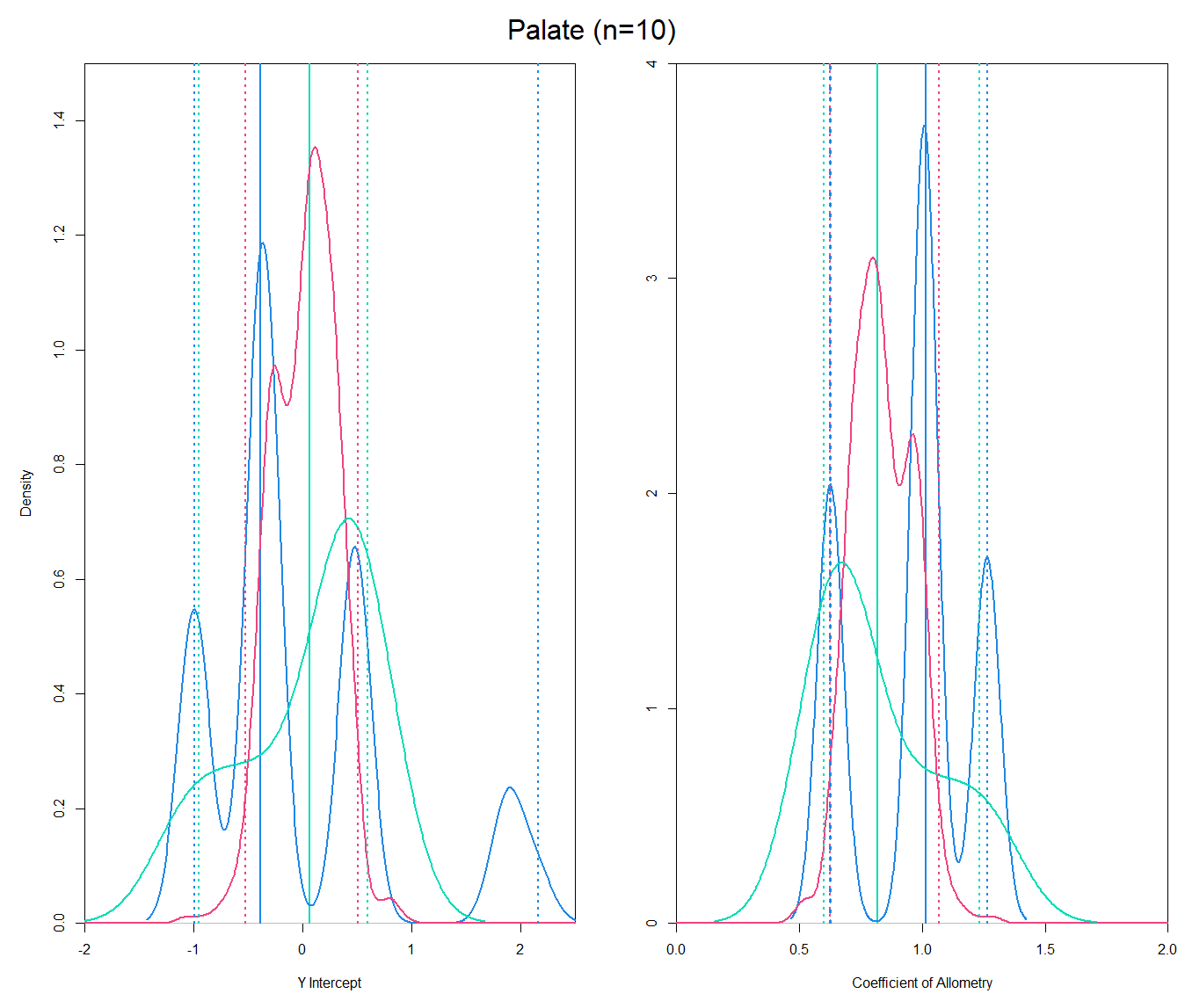


**Fig S4:** Bootstrapping palate length against total skull length in *Exaeretodon argentinus*. Cyan=generalized linear mixed model; Blue=ordinary least squares regression on undistorted data; Red=ordinary least squares regression on distorted data; and Dashed lines=95% confidence intervals; solid lines=recovered y-intercept and coefficient of allometry from analyses on the full dataset. Skull is specimen MCZ VPRA-4470.


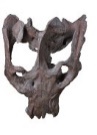

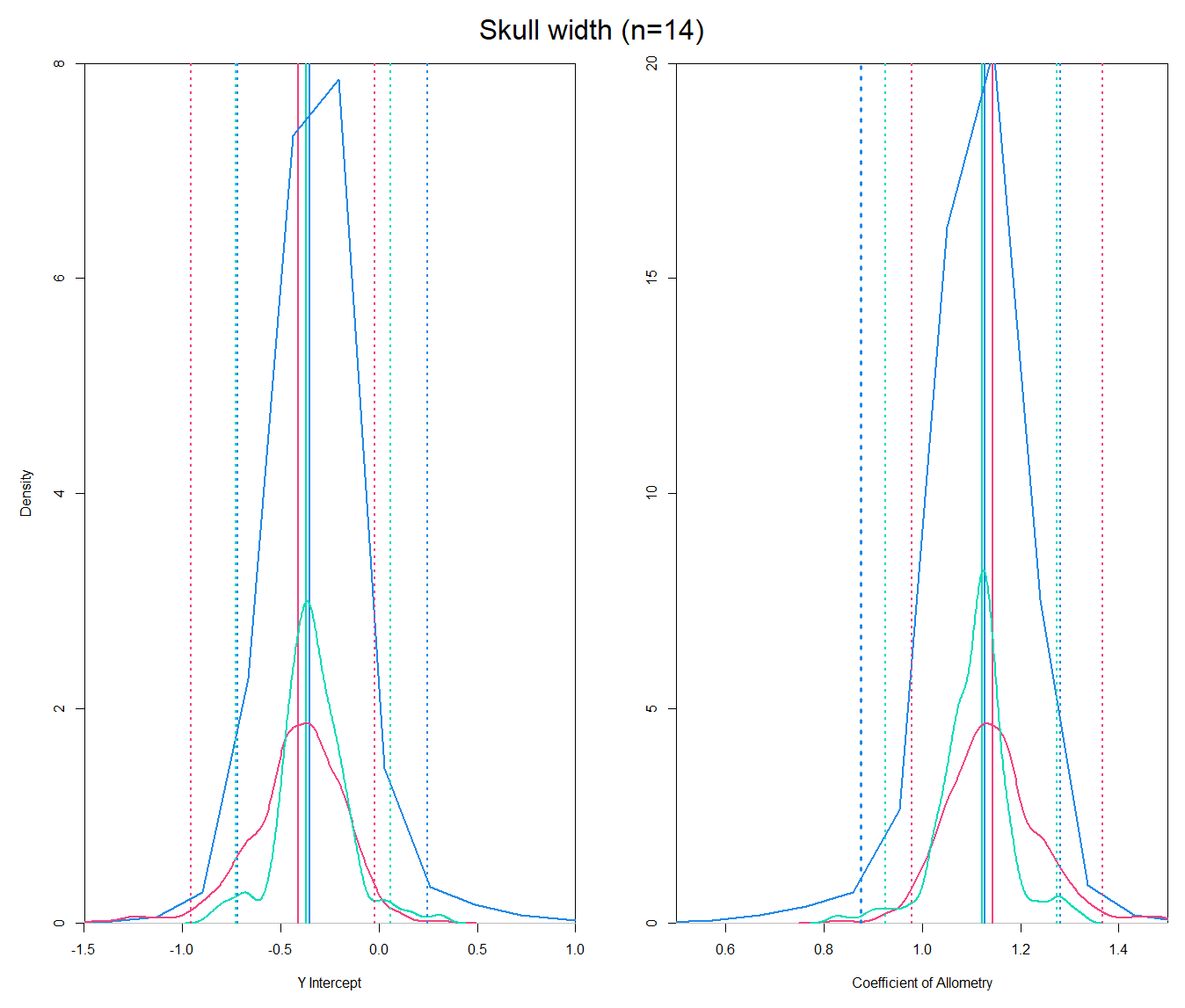


**Fig S5:** Bootstrapping skull width against total skull length in *Exaeretodon argentinus*. Cyan=generalized linear mixed model; Blue=ordinary least squares regression on undistorted data; Red=ordinary least squares regression on distorted data; and Dashed lines=95% confidence intervals; solid lines=recovered y-intercept and coefficient of allometry from analyses on the full dataset. Skull is specimen MCZ VPRA-4470.


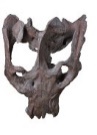

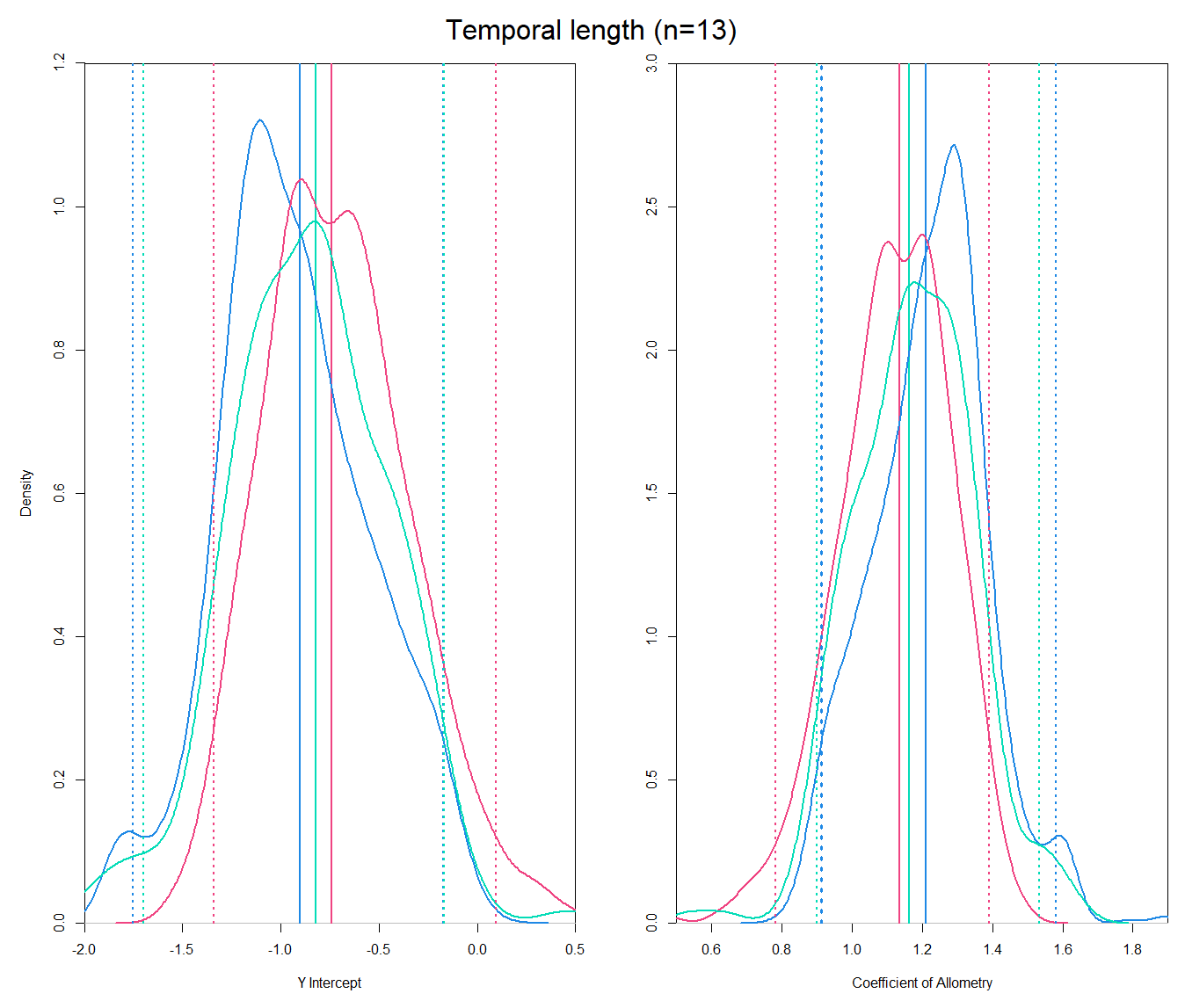


**Fig S6:** Bootstrapping temporal length against total skull length in *Exaeretodon argentinus*. Cyan=generalized linear mixed model; Blue=ordinary least squares regression on undistorted data; Red=ordinary least squares regression on distorted data; and Dashed lines=95% confidence intervals; solid lines=recovered y-intercept and coefficient of allometry from analyses on the full dataset. Skull is specimen MCZ VPRA-4470.

**Table S3:** Summary statistics for generalizd linear mixed model of muzzle length

|  | **Exa_data$MUL** | | |
| --- | --- | --- | --- |
| *Predictors* | *Estimates* | *CI* | *p* |
| (Intercept) | -0.27 | -0.56 – 0.01 | 0.058 |
| Exa_data$BSL | 0.96 | 0.85 – 1.08 | **<0.001** |
| **Random Effects** | | | |
| σ^2^ | 0.00 | | |
| τ_00_ _I_ | 0.00 | | |
| ICC | 0.02 | | |
| N _I_ | 14 | | |
| Observations | 22 | | |
| Marginal R^2^ / Conditional R^2^ | 0.926 / 0.927 | | |
