# Supplementary material for: Including Distorted Specimens in Allometric Studies: Linear Mixed Models Account for Deformation
Source: Integr Org Biol. 2021 May 18;3(1):obab017. doi: 10.1093/iob/obab017 (PMC8341891; doi:10.1093/iob/obab017)
Supplement: obab017_Supplementary_Data [file obab017_supplementary_data.zip › obab017_Supplementary_Data/Wynd_Abstract_Spanish.docx]

Alometria– el estudio de patrónes midiendo los cambios de proporciónes entre diferentes partes del cuerpo–es un método popularmente usado para estudiar como clados exhiben patrónes fenotípicos que representan restricciónes evolutivas, o para cuantificar patrónes de ontogenia entre una especie. Reconstruyendo alometrias para series ontogeneticas es uno de los pocos métodos disponibles para reconstruir el crecimiento de especies fósiles. Sin embargo, fósiles sufren de deformaciónes tafonomicas que alteran la morfología original y algunas veces en maneras no deseadas. Para mitigar estas alteraciones tafonomicas, paleontólogos excluyen mediciones alteradas de sus análisis. Desafortunadamente, esto limita el numero de muestras y potencialmente elimina evidencia de variación individual, impactando reconstrucciones alometricas. Mínimos Cuadrados Ordinarios (MCO) es un método frecuentemente usado para estimar alometria, pero asume niveles constantes de varición entre especímenes; esto es improbable cuando uno incluye especímenes deformados y especímenes indeformables. Alternativamente, Modelos Lineales Generalizados Mixtos (MLGM) pueden atribuir variciónes adicionales en un modelo. Nosotros corrimos simulaciones basadas en análisis empíricos del cinodonte extinto, *Exaeretodon argentinus*, para determinar la eficacia de MLGM con datos alometricos. Nosotros descubrimos que MLGM estima la alometria usando un conjunto de datos completos, en lugar de solo usar datos distorsionados. Aplicamos este método en dos conjuntos de datos empíricos: medidas craneales de especímenes de *E. argentinus* (n = 16) y medidas femorales del dinosaurio *Tawa hallae* (n = 26). Nuestros estudios indican que MLGM puede reconstruir mejor los patrónes de alometria sobre MCO con conjuntos de datos que incluyen especímenes extintos, y debería ser el protocolo estándar cuándo se usan especímenes que están deformados.
